# Single photon emission computed tomography (SPECT) of anxiety disorders before and after treatment with citalopram

**DOI:** 10.1186/1471-244X-4-30

**Published:** 2004-10-14

**Authors:** Paul D Carey, James Warwick, Dana JH Niehaus, Geoffrey van der Linden, Barend B van Heerden, Brian H Harvey, Soraya Seedat, Dan J Stein

**Affiliations:** 1 MRC Unit on Anxiety Disorders, Department of Psychiatry, University of Stellenbosch, Tygerberg, 7505, Cape Town, South Africa; 2 Department of Nuclear Medicine, University of Stellenbosch, Tygerberg, 7505, Cape Town, South Africa; 3 School of Pharmacy (Pharmacology), North-West University, Potchefstroom, South Africa

## Abstract

**Background:**

Several studies have now examined the effects of selective serotonin reuptake inhibitor (SSRI) treatment on brain function in a variety of anxiety disorders including obsessive-compulsive disorder (OCD), posttraumatic stress disorder (PTSD), and social anxiety disorder (social phobia) (SAD). Regional changes in cerebral perfusion following SSRI treatment have been shown for all three disorders. The orbitofrontal cortex (OFC) (OCD), caudate (OCD), medial pre-frontal/cingulate (OCD, SAD, PTSD), temporal (OCD, SAD, PTSD) and, thalamic regions (OCD, SAD) are some of those implicated. Some data also suggests that higher perfusion pre-treatment in the anterior cingulate (PTSD), OFC, caudate (OCD) and antero-lateral temporal region (SAD) predicts subsequent treatment response. This paper further examines the notion of overlap in the neurocircuitry of treatment and indeed treatment response across anxiety disorders with SSRI treatment.

**Methods:**

Single photon emission computed tomography (SPECT) using Tc-^99 m ^HMPAO to assess brain perfusion was performed on subjects with OCD, PTSD, and SAD before and after 8 weeks (SAD) and 12 weeks (OCD and PTSD) treatment with the SSRI citalopram. Statistical parametric mapping (SPM) was used to compare scans (pre- vs post-medication, and responders vs non-responders) in the combined group of subjects.

**Results:**

Citalopram treatment resulted in significant deactivation (p = 0.001) for the entire group in the superior (t = 4.78) and anterior (t = 4.04) cingulate, right thalamus (t = 4.66) and left hippocampus (t = 3.96). Deactivation (p = 0.001) within the left precentral (t = 4.26), right mid-frontal (t = 4.03), right inferior frontal (t = 3.99), left prefrontal (3.81) and right precuneus (t= 3.85) was more marked in treatment responders. No pattern of baseline activation distinguished responders from non-responders to subsequent pharmacotherapy.

**Conclusions:**

Although each of the anxiety disorders may be mediated by different neurocircuits, there is some overlap in the functional neuro-anatomy of their response to SSRI treatment. The current data are consistent with previous work demonstrating the importance of limbic circuits in this spectrum of disorders. These play a crucial role in cognitive-affective processing, are innervated by serotonergic neurons, and changes in their activity during serotonergic pharmacotherapy seem crucial.

## Background

Significant advances in our understanding of the mediating psychobiology and the development of effective treatments for anxiety disorders have been made in recent years. Modern brain imaging techniques have proved useful in exposing specific albeit overlapping neurocircuitry that underlies individual anxiety disorders [1,2]. However, relatively little work has focused on the extent to which the anxiety disorders overlap with respect to changes in brain perfusion that accompany response to first-line treatment that is after all pharmacologically similar for different disorders.

The selective serotonin reuptake inhibitors (SSRIs) are currently recommended as first line medications for most anxiety disorders, including obsessive-compulsive disorder (OCD) [3], posttraumatic stress disorder [4] and social anxiety disorder [5]. A number of imaging studies have now examined the effects of SSRI's on brain perfusion in individual anxiety disorders. In OCD, attenuation of pre-treatment regional activation has been shown to correlate with treatment response in the anterolateral orbitofrontal cortex (OFC), caudate nucleus, thalamus, and temporal regions [6-11]. Results for studies assessing pre-treatment cerebral perfusion as a predictor of response, have, however, yielded mixed results. In some, an inverse relationship appears to exist with pre-treatment regional activation of the OFC [12], anterior cingulate, caudate [6] and subsequent responses to treatment. Conversely findings of higher prefrontal, cingulate and basal ganglia activation correlating with subsequent treatment response have also been reported [13,14]. In OCD co-morbid with depression, substrates of response to the SSRI, paroxetine, appear to differ based on pretreatment [15] activation patterns as well as changes that accompany treatment response when an SSRI is given in identical doses for either of the two conditions separately [16].

In social anxiety disorder SSRI treatment response accompanies attenuation of frontal, anterior and lateral temporal cortex, cingulate, and thalamic activity [17,18]. Higher anterior and lateral temporal cortical perfusion at baseline correlated with subsequent treatment response in the former study. The latter study also demonstrated some overlap of regions demonstrating attenuation of activity for both cognitive and pharmacotherapy interventions.

In PTSD, a single study by our group demonstrated medial temporal lobe deactivation with treatment irrespective of clinical response and medial prefrontal cortex activation correlated with treatment response. In addition, no baseline differences distinguished responders and non-responders to subsequent SSRI treatment [19].

In this present study, we hypothesised firstly, that response to SSRI treatment in this combined group of subjects with anxiety disorders (OCD, PTSD, SAD) would effect shared changes in rCBF affecting primarily limbic and related prefrontal regions and thus suggest some overlap between disorders in the mechanism of their response to effective treatment with SSRI's. Secondly, pre-treatment differences in regional perfusion would likely differentiate responders to subsequent treatment with citalopram across the anxiety disorders.

## Methods

### Subjects

Adult subjects with a primary diagnosis of OCD (n = 11), PTSD (n = 11) or SAD (n = 15) were recruited from the Anxiety Disorders Clinic of our tertiary hospital. All subjects were interviewed with the Structured Clinical Interview for the Diagnosis of Axis-I Disorders [20] to ascertain diagnosis according to DSM-IV criteria. Results for the PTSD group have been reported previously [19].

Comorbid major depression was an exclusion criterion in the OCD and SAD, but not in the PTSD subjects. Nevertheless, in all cases comorbid disorders were considered secondary in terms of temporal course, symptom severity, and associated distress. Patients previously treated with SSRI's had been free of medication for a minimum of four weeks for fluoxetine and two weeks for other SSRI's. In total 30 (81%) of the group were SSRI naïve. Subjects with other central nervous system disorders including previous head injury or epilepsy were excluded. The Institutional Review Board of our University approved the protocol and all patients gave informed written consent after a full explanation of the possible risks and benefits.

### Pharmacotherapy and measures

All patients underwent treatment with citalopram, the most selective of the currently available selective serotonin reuptake inhibitors (SSRIs). The duration of the trial of treatment was 12 weeks for OCD and PTSD, and 8 weeks for SAD.

Dosage was initiated at 20 mg daily for the first two weeks and then maintained at 40 mg daily for the remainder of the study. Measures of symptom improvement were made bi-weekly by clinicians using the Clinical Global Impressions (CGI) scale [21]. Subjects with a CGI change score of 2 or less post-treatment were defined as responders, while those with scores greater than 2 were defined as non-responders.

Anxiety symptoms were also rated using disorder specific scales including the Liebowitz Social Anxiety Scale(LSAS) [22], the Yale Brown Obsessive-Compulsive Scale (YBOCS) [23] and the Clinician Administered Scale for PTSD [24]. Depressive symptoms were rated using the Montgomery-Asberg Depression Rating Scale (MADRS) [25].

### SPECT imaging

Single photon emission computed tomography (SPECT) was conducted before and after pharmacotherapy. Subjects lay supine in a quiet dimly lit room for 30 minutes prior to injection of the radiopharmaceutical. Apart from administration of the injection by a physician, they remained alone in the room during this period. Subjects were asked to remain at rest during the 30 minute period and for 10 minutes after injection of the radiopharmaceutical.

An injection of 555 MBq (15 mCi) of technetium-99 m hexamethylpropylene amine oxime (Tc-99 m HMPAO) was given into an arm vein through a previously placed intravenous cannula. After completion of the rest period, SPECT imaging of the brain was performed, with the subject's head supported by a headrest, using a dual detector gamma camera (Elscint, Helix, GE Medical Systems, USA) equipped with fan beam collimators.

Data were acquired in the step-and-shoot mode, using a 360 degree circular orbit, with the detectors of the gamma camera as close as possible to the subject's head. The height of the imaging table and radius of rotation were noted for each subject and the same measurements were used for the follow-up study. Data were acquired using a 128 × 128 image matrix in 3 degree steps of 15 seconds per step.

Data were reconstructed by filtered backprojection, using a Metz filter (power = 5, FWHM = 14 mm) and a zoom factor of 2.29. The Chang (1978) method was used for attenuation correction. Scatter correction was not performed. The final reconstructed pixel size was 3.87 mm by 3.87 mm. Image files were converted from interfile to analyze format using conversion software (Medcon, Erik Nolf, UZ Ghent).

Stastical analyses were conducted on a voxel-by-voxel basis using the Statistical Parametric Mapping (SPM99, Wellcome Department of Cognitive Neurology, UK) [26]. The realign function was used to co-register baseline and posttreatment SPECT images for each subject and to generate a mean image for each subject. Realigned images were then normalised to the Montreal Neurological Institute (MNI) standard anatomical space to a value of 50 using proportional scaling. For this the transform function from the mean image for each subject to the normalised image with 4 mm^3 ^voxels using 12 affine transformations and 7 × 8 × 7 non-linear basis functions was used. Standardised images were then smoothed using a Gaussian kernel with a FWHM of 12 mm^3^.

A multi-group study design was performed using 2 groups (responders and non-responders) with 2 conditions each (pre- and post-treatment). Contrasts were applied to look for areas of significant change post-treatment compared to pre-treatment. Contrasts were also used to search for areas of relative change in treatment responders compared to non-responders. A second design was employed to compare the baseline scans of responders to SSRI pharmacotherapy with those of non-responders. Contrasts were used to search for regions of significant differences on the baseline scans of responders compared to non-responders.

In view of *a priori *knowledge suggesting involvement of the cingulate, hippocampus, inferior frontal cortex, and striatum in the anxiety disorders, an uncorrected p-value of p < 0.001 corresponding to a t value of 3.34, was chosen for the analysis of these regions in order to minimize type I errors. Given the relative paucity of data in this area, we chose this uncorrected p-value, based on work using a similar methodology [19]. In order to minimize type I errors a significance level of p < 0.05 corrected for Gaussian Random Field Theory was used for the remainder of the brain. A spatial extent threshold of 5 voxels was also used at all times. Masking using a threshold proportional to 0.4 times the mean voxel value was used to minimize the analysis of voxels not located in grey matter. Furthermore, clusters were ignored if co-registration with a SPECT template demonstrated that they were located outside of grey matter.

## Results

Twenty-two males and fifteen females with a mean age of 33.5 years (SD 9.8) completed the study. Clinical changes with pharmacotherapy for each disorder are provided in Table 1. This shows that for each of the anxiety disorders being studied, citalopram was effective in significantly reducing clinical measures of severity as determined by a CGI change score of 2 or less (much or very much improved). As such, 20 of 37 patients (54%) were responders to citalopram.

**Table 1:** Clinical parameters for all the groups (mean ± SD), (paired t-test).

		**Baseline**	**Endpoint**	**p**
OCD (n = 11)	YBOCS	26.6 ± 4.7	23.7 ± 5.8	0.001
	MADRS	13.64 ± 9.6	9.9 ± 6.4	0.119
	CGI-severity	4.7 ± 0.647	4.18 ± 1.1	0.025
	CGI-improvement		3.1 ± 0.7	
SAD (n = 15)	LSAS	79.2 ± 30.2	63.1 ± 28.5	0.003
	MADRS	15 ± 4.9	9.1 ± 5.9	0.004
	CGI-severity	4.6 ± 0.8	3.3 ± 1.1	0.001
	CGI-improvement		2.7 ± 1.2	
PTSD (n = 11)	CAPS	78.1 ± 16.9	45.5 ± 23.9	<0.01
	MADRS	25 ± 6.7	15.9 ± 8.0	<0.01
	CGI -severity	4.5 ± 0.5	2.5 ± 0.7	<0.01
	CGI-improvement		1.9 ± 0.7	

YBOCS, Yale Brow Obsessive-compulsive scale; MADRS, Montgomery Asberg Depression Rating scale; CGI-s, Clinical global impressions severity; CGI-I, Clinical global impressions – improvement; LSAS, Liebowitz Social Anxiety Scale; CAPS, Clinician Administered PTSD scale.

**Figure 1:**
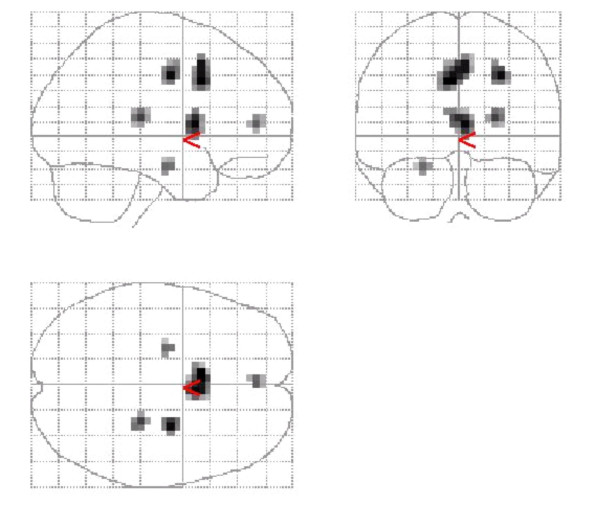
**Combined group deactivation following treatment with citalopram. **Regions of deactivation for the combined group of OCD + SAD + PTSD following treatment with citalopram. Significant grey matter clusters are seen in the superior cingulate, anterior cingulate, left medial temporal region (hippocampus).

Comparison of pre- and post-treatment scans for the whole group showed decreased activity in 4 significant clusters in grey matter (Figure 1): These included the superior cingulate, right thalamus, anterior cingulate, and the left hippocampus (Table 2). Comparison of pre- and post-medication scans showed no significant areas of activation.

**Table 2:** Localisation of significant clusters of deactivation following treatment for the combined group of OCD, SAD, PTSD. Z_max _set to threshold of t = 3.34 corresponding to p < 0.001

Cluster size (voxels)	t	MNI co-ordinates (x,y,z)	Brain region
44	4.78	-4,12,36	Superior cingulate
19	4.66	24,-28,12	Right thalamus
10	4.04	0,48,8	Anterior cingualate
7	3.96	-24,-12,-20	Left hippocampus

**Figure 2:**
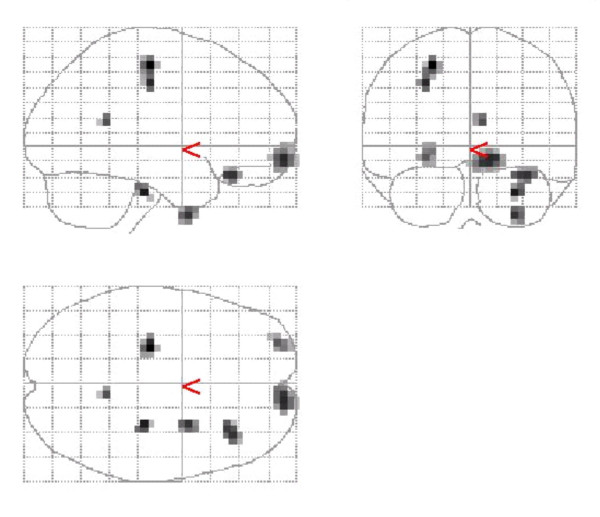
**Regional deactivation (responders > non-responders). **Grey matter clusters of greater deactivation in responders vs non-responders were detected in the left precentral, prefrontal and right mid - and inferior-frontal regions.

Comparison of responders with non-responders demonstrated that responders had a significantly greater decrease of activity in 4 clusters (Figure 2). These clusters were localised to the left precentral, right middle frontal, right inferior frontal and, left prefrontal regions (Table 3). Comparison of baseline scans of responders and non-responders did not reveal any significant differences.

**Table 3:** Localisation of significant clusters of deactivation in responders vs non-responders to SSRI treatment for the combined group of OCD, SAD, PTSD. Z_max _set to threshold of t = 3.34 corresponding to p < 0.001

Cluster size (voxels)	t	MNI co-ordinates (x,y,z)	Brain region
21	4.26	-24,-20,56	Left precentral
33	4.03	12,64,-8	Right mid-frontal
17	3.99	36,32,-20	Right inferior frontal cortex
5	3.85	8,-48,16	Right precuneus
18	3.81	-28,60,-8	Left prefrontal

## Discussion

The main finding of this paper was that citalopram pharmacotherapy resulted in significant deactivation within anterior and superior cingulate cortex, the left hippocampus and the right thalamus in a combined group of patients with different anxiety disorders (OCD, PTSD, and SAD). Furthermore, deactivation was significantly more apparent in responders than in non-responders to SSRI treatment within precentral, right inferior, middle frontal and left prefrontal regions. Interestingly, no pre-treatment differences in regional perfusion between subsequent treatment responders vs non-responders were found.

Although there are important differences in the symptomatology of the anxiety disorders, these conditions do share certain aspects of their phenomenology, including heightened anxiety and avoidance behaviour. Furthermore, previous functional brain imaging work has demonstrated overlapping neurocircuitry across different anxiety disorders with activation of paralimbic circuitry and right inferior frontal cortex in a combined group comprising subjects with OCD, PTSD, and specific phobia [1]. Results in the present study now also point to an overlap in the functional neuroanatomy, primarily implicating paralimbic neurocircuitry, in treatment response to the same SSRI, citalopram, across anxiety disorders. In citalopram responders, effects across disorders were most pronounced in the mid, inferior and prefrontal cortex. In other regions, such as the striatum, data on treatment response and symptom provocation seems to indicate less overlap across anxiety disorders, which may suggest only partial and regionally specific overlap between disorders [1,2].

Specific limbic regions are well-known to play a role in broadly mediating anxiety. Early observations of epileptogenic cingulate lesions support its role in regulating affect [27]. Furthermore, recent work has suggested a role for the anterior cingulate in integrating cognitive and motivational processes. These include evaluating environmental cues and monitoring performance [28] On the other hand, a central role for the hippocampus in contextual aspects of fear conditioning has been demonstrated [29,30].

The findings here complement previous studies of OCD, PTSD, and SAD that have demonstrated a specific role for the cingulate and hippocampus in these conditions. Studies in OCD have shown increased anterior cingulate activity at baseline, or deactivation during pharmacotherapy with serotonergic agents [31]. In PTSD, anterior cingulate activity is also increased in some, although not all, studies of PTSD [32,33] Further, the anterior cingulate is deactivated during citalopram treatment of SAD patients [17]. Dysfunction of the hippocampus, as indicated by smaller hippocampal volume and declarative memory deficits, may play an important role in PTSD [34].

The medial prefrontal cortex comprises several related areas including anterior cingulate cortex. Lesions of this area are associated with suboptimal responses to stress, and the area has important inhibitory inputs to the amygdala which mediate extinction of fear conditioning [29]. The middle and inferior frontal cortex, on the other hand, is involved in encoding and retrieval of verbal memories. Our finding that the right inferior frontal cortex was more deactivated in responders is perhaps consistent with previous findings showing increased activity pre-treatment in this region across different anxiety disorders [2] and in some, but not all, studies of PTSD [35].

Serotonergic circuits innervate the medial prefrontal cortex and other limbic structures, and chronic administration of a serotonin reuptake inhibitor may lead to an increase in their neurotransmission. It is possible that the medial prefrontal cortex deactivation during serotonergic pharmacotherapy indicates that a compensatory increase of activity in this region is no longer needed after symptom improvement. Along these lines, a number of functional and electrophysiological imaging studies of depression have found that anterior cingulate hyperactivity predicts a positive response to pharmacotherapy, a finding that has also been interpreted as indicating the baseline presence of an adaptive compensatory response [36]. In addition changes in cognitive processing of frontal cortex may be secondary to symptom reduction caused by primary drug-induced changes within the limbic system. We have previously demonstrated similarly higher pre-treatment prefrontal perfusion in subsequent responders relative to non-responders using inositol in OCD [37]. Interestingly, inositol responsive disorders overlap with those responsive to SSRI's which may suggest that it is serotonergic components of these disorders that account for at least some of the overlap in perfusion patterns demonstrated here.

In contrast, however, increased activity in anterior cingulate or orbitofrontal region in OCD has also been shown to predict a poorer response to pharmacotherapy [9].

Perhaps increased activity in particular limbic circuits plays a different functional role in different psychiatric disorders. Only limited functional imaging studies of pharmacotherapy effects have involved provocation paradigms [38] and such differences in design may account for certain inconsistencies across studies. Alternatively, it is feasible that different effects in different disorders may also help explain inconsistencies. In the current dataset, however, we were unable to demonstrate any associations between baseline activity and pharmacotherapy response for the combined group.

This study is limited by the slightly different inclusion criteria (inclusion of secondary depression in PTSD group) and pharmacotherapy duration for different disorders. While the absence of untreated controls may to some extent limit the conclusions we can draw, comparing non-responders to responders we believe serves as a reasonable evaluation of changes that result from treatment response. The lower spatial resolution of SPECT may be considered a limitation nevertheless this study usefully emphasizes the importance of limbic regions (amygdala, hippocampus) in mediating anxiety. Furthermore, deactivation within these regions as well as richly connected frontal regions following SSRI treatment, particularly in responders, is clearly demonstrated. Further research combining pharmacological interventions and functional methodologies, and using tracers tailored to specific neurotransmitter receptors, will undoubtedly lead to increased understanding of the pathogenesis of the anxiety disorders and the mechanisms of response to treatment in the future.

## Competing interests

The authors declare that they have no competing interests.

## Authors' contributions

The authors contributed to the work in the following ways: drafting of manuscript (PDC, JW, DJS), data analysis (JW, PDC), psychiatric evaluations (DJHN, GVDL, SS, DJS), SPECT imaging (JW, BBVH). All authors have read and approved the final manuscript.

## Pre-publication history

The pre-publication history for this paper can be accessed here:


